# Supplementary material for: The vicious circle of stereotypes: Teachers’ awareness of and responses to students’ gender-stereotypical behaviour
Source: PLoS One. 2022 Jun 15;17(6):e0269007. doi: 10.1371/journal.pone.0269007 (PMC9200163; doi:10.1371/journal.pone.0269007)
Supplement: Attachment — Submitted filename: Review PlosONe.pdf [file pone.0269007.s001.pdf]

Review PlosONe: The Vicious Circle of Stereotypes. Teachers' Awareness of and Responses to Students' Gender-Stereotypical Behaviour

Thank you for the opportunity to review this paper. The data that are gathered are impressive and the mixed method combination fits the goals of the paper. I think the topic is very relevant in light of boys' underperformance in education, gender segregation in education and other problems that arise from the reinforcement of gender stereotypes in schools. Although there were parts of the paper that I liked, I think there is also room for improvement. My comments can be found below, the most important ones are in bold.

Abstract

Please mention in abstract what type of education is studied (primary/secondary school).

Introduction/theory

- "Despite the passage of time, these social beliefs about gender roles have remained remarkably stable" → do you have a more recent reference?
- **I miss a) why we want to know this (some reasons are touched upon on page 13 but this should come sooner/be worked out more), b) what has previous research found regarding the questions you ask/goals you set c) linking to b: what is the innovation of this study?**
- I miss references to key works the social construction of gender such as West & Zimmerman (1987). Also: take a look at the work of Mieke van Houtte.
- In the part on biological differences between girls/boys the conclusion is drawn that both biological/social factors should be taken into account. How is that done in this study?
- I would be careful with referring to Gurian and Sommers as evidence for the fact that brains differ between girls and boys; there are also studies that show there are very little differences or that brain differences actually emerge from social inequalities. At least mention this is not an undisputed fact. See e.g. these works:

1. Wierenga, L. M., Bos, M. G., van Rossenberg, F., & Crone, E. A. (2019). Sex effects on development of brain structure and executive functions: greater variance than mean effects. Journal of cognitive neuroscience, 31(5), 730-753.
2. Kleinherenbrink, A. (2014). Mapping plasticity: Sex/gender and the changing brain. Tijdschrift voor Genderstudies, 17(4), 305-326.

- **In sum: The part before "the present study" is a quite unstructured literature review. It has some nice parts, but I really miss a logical and concise build-up to certain expectations regarding the goals over the paper. E.g. there is quite some elaboration about concepts that are not directly and/or clearly related to the goals of the study.**

Present study/method

- **I would like more explanation of why the specific these forms of data gathering were used. What is the advantage of doing observations/interviews (in light of your goals)? What questions were answered by observations and what questions by interviews? I sometimes miss the clear link between introduction (goals), theory (see previously: what are the expectations based on previous studies and theory), method (what method is used for what question/goal/expectation).**

- Some reflection on how teacher's behaviour might be altered because of a) the presence of the researcher and b) knowledge about what the study was about (it is not mentioned what information was given to the teacher before observation).
- I am not a qualitative researcher but the (method of) analysis seems thorough and sound.

Results

- **As said, I am not a qualitative researcher but I think the result section is far too long and should be more focussed. In total it's 18 pages (there are full articles that are shorter) and there are 7 pages alone on describing behaviour patterns that differ between girls and boys (observed by teachers). I appreciate the authors wanting to present all these interesting quotes, but it makes the paper not well readable and makes the reader lose track of what the goals are and how these qualitative results add to these goals.**
- Ways to shorten and structure the result section: There are often multiple quotes to illustrate 1 point and some quotes are very long (e.g. 3 long quotes on page 27). There are also quotes that do not add to the text at all (eg. the quote on the intimate relation with the teacher, what is the goal/context of this quote?). General conclusions (sh/)could be drawn at the end of a section so that the link to the goal is clear. My suggestion would be to seriously reduce the number of quotes, focus the text so that it clearly relates to the goals of the study (e.g. draw clear conclusions about the different goals at the end of the result sections).

Conclusion

- **Most importantly: I miss a structured reflection on the 4 goals of the paper.**
- Could an explanation for boys receiving more attention from the teacher be that they are underperforming (and teachers spend more time on underperforming students)?
- Small: this sentence is unclear: "Meanwhile, in order for teachers' interventions to be effective, they should relate, and more specifically, break the vicious circle of stereotypes (Pankowska, 2005) or show a different strategy of breaking stereotypical behaviour among students." → for teachers interventions to be effective (to break gender stereotypes?) they should break the vicous circle of stereotypes?
- I would not spend a substantial part of the conclusion introducing a theoretical concept ("activation of a negative stereotype about the low ability level of an individual's own group").
- There is mentioning on changes in education in Poland but these are not explained. What is meant by this? : "The situation of education in Poland since the beginning of the nineties of the last century has constantly been reformulated (Jakubowski, 2021), and the recent ministerial changes initiated with the introduction of another education reform in 2017 are revolutionary rather than evolutionary (Ministry of Education and Science, 2021)."
- There could be more suggestions for future research
- Would the fact that you only surveyed female teachers have influenced the findings? There could be some reflection on this.
