# Supplementary material for: The vicious circle of stereotypes: Teachers’ awareness of and responses to students’ gender-stereotypical behaviour
Source: PLoS One. 2022 Jun 15;17(6):e0269007. doi: 10.1371/journal.pone.0269007 (PMC9200163; doi:10.1371/journal.pone.0269007)
Supplement: Attachment — Submitted filename: Response to Reviewers.docx [file pone.0269007.s003.docx]

| **First Reviewer's Comments** | |
| --- | --- |
| **Comment 1:** I thank the authors for their thorough revision of the manuscript and their answers to my comments. In my opinion, the manuscript has significantly improved. However, I still have some remarks: | **Response 1:** We would like to thank the reviewer for this comment. This time also we will do our best to improve the article. |
| **Comment 2:** In the abstract, I would change "a self-fulfilling prophecy" to "self-fulfilling prophecies". | **Response 2:** The change was introduced as suggested by the reviewer. |
| **Comment 3:** In the section about sex and gender the authors define sex as "anatomical and psychological features" - I don't agree with that. Also I wonder about the reference here. Why don't you cite Kay Deaux or West & Zimmerman? | **Response 3:** The sentence “anatomical and psychological features” has been removed from the section. We would like to note that West & Zimmerman is quoted in this paragraph as suggested in a previous round of reviews (item 11: West C, Zimmerman, DH. Doing gender. Gender & society. 1987; 1 (2): 125-151) while one of Kay Deaux's books is also quoted (item 4 in reference list: Kite M, Deaux K, Haines EL. Gender stereotypes. In: Denmark FL, Paludi MA, editors. Psychology of women: A handbook of issues and theories. Santa Barbara, CA: Praeger an imprint of ABC-CLIO, LLC; 2017. p. 205-36.) |
| **Comment 4:** I would add agency/communion (or instrumentality/expressivity) as the theoretical dimensions behind gender stereotypical attributes, see e.g. Kachel et al. (2016) or Abele & Wojciszke (2007) | **Response 4:** As suggested by the reviewer, the paragraph on stereotypical male and female features was supplemented with the proposed dimensions. |
| **Comment 5:** I suggest to change the order of the research questions so that it matches the logic of the paper. So first pose the question that is answered through observation and then add the questions that are answered through the interviews. Moreover, there shouldn't be a RQ 3.1. if there is no 3.2. | **Response 5:** As suggested by the reviewer, the order of the research questions was changed to better correspond to the order in which the research results were presented. |
| **Comment 6:** Please add sample statements to all categories presented in the Results & Discussion section (e.g. Biological / psychological factors) | **Response 6:** Where necessary, quotes from teachers' statements were added to the analytical categories. Thank you for highlighting this element. We missed this issue when shortening and reformulating the previous version of the text. |
| **Comment 7:** References to the literature from the introduction section are missing from the Results & Discussion Section. The authors should decide if they want a pure Results section (without references) or a Results and Discussion section than really discusses the results with regard to the literature. | **Response 7:** Thank you for this comment, we decided to leave the Results section pure and move the discussion to the end of the article, with references to the literature. |
| **Comment 8:** In the Conclusion section, I suggest to change the wording here "The presented study was designed to verify; (i) teachers' awareness of gender stereotyped behaviours of girls and boys, (ii) teachers' awareness of the possible causes of these behaviours, (iii) teachers' actions to respond to these behaviours, including those that may deepen gender bias and gender stereotypes, and (iv) teachers' awareness of the existence of gender-polarized content of school textbooks." In my opinion, it's not about verifying but about investigating. | **Response 8:** Thank you for highlighting this point. We agree, the proposed change has been made. |
| **Comment 9:** Please mention existing interventions that could improve the situation, e.g. Kollmayer et al. (2020) | **Response 9:** Thank you for your suggestion to mention existing interventions. In line with this commentary, the necessary addition has been made to the text. |
| **Comment 10:** Moreover, I proof-reading the whole manuscript for language and spelling errors and sentences that got mixed up during the revision (especially in the Results & Discussion section). | **Response 10:** The article was returned to proofreading. We hope that in its current version it meets the stylistic and grammatical requirements. |
